# Bronchopneumonia

**Published:** 1918-11

**Authors:** 


***Bronchopneumonia.*** By W. G. MacCallum, Baltimore. *Journal American Medical Association*, Aug. 31, 1918.

The author reports the results of his study of an epidemic of broncho-pneumonia at Camp Dodge, Iowa, where the surroundings and environment generally were very different from those attending the bronchopneumonia epidemic previously reported by him at Camp Houston, Texas. There were no great numbers of measles patients at Camp Dodge, and methods of isolation and sterilization were thorough in the hospital. The great spread of infection appeared to be in the camp itself. During the winter there was a moderate amount of pneumococcus pneumonia with low mortality, but on March 20 this mild pneumonia was replaced by a severe disease which began in an organization of Alabama negroes and spread rapidly to other portions of the camp. Throughout this epidemic, pneumococcus infections resulting in lobar pneumonia continued to appear. From the whole, the author thinks it justifiable to hold that *streptococcus haemolyticus*, whether preceded by predisposing disease like measles, or not, may cause extensive and fatal epidemics of a peculiar bronchopneumonia, usually affecting the framework of the lungs and the bronchial walls so as to suggest the name interstitial bronchopneumonia, especially severe if following measles. Associated with it there is often a diffuse or patchy lobular pneumonia with the streptococcus finely scattered in the alveolar exudate, and when such areas are confluent they may resemble a lobar pneumonia. Ulcerated laryngitis, possibly causing deep destruction of the vocal cords, and epiglottis occur in the more acute cases, especially after measles. Empyema is also extremely common and has been generally recognized, but other complications are not so prominent. Pericarditis, peritonitis, otitis media, and occasional abscess are mentioned. Hyaline degeneration of the muscle fibers of the rectus abdominis, quite similar to the Zenker's degeneration in typhoid fever, was observed in one case in which it caused rupture of the muscle. It seems to have been due to an extension of the infection in the degenerated muscle.

